# Framing the discussion of microorganisms as a facet of social equity in human health

**DOI:** 10.1371/journal.pbio.3000536

**Published:** 2019-11-26

**Authors:** Suzanne L. Ishaq, Maurisa Rapp, Risa Byerly, Loretta S. McClellan, Maya R. O’Boyle, Anika Nykanen, Patrick J. Fuller, Calvin Aas, Jude M. Stone, Sean Killpatrick, Manami M. Uptegrove, Alex Vischer, Hannah Wolf, Fiona Smallman, Houston Eymann, Simon Narode, Ellee Stapleton, Camille C. Cioffi, Hannah F. Tavalire

**Affiliations:** 1 Biology and the Built Environment Center, University of Oregon, Eugene, Oregon, United States of America; 2 Robert D. Clark Honors College, University of Oregon, Eugene, Oregon, United States of America; 3 Department of Human Physiology, University of Oregon, Eugene, Oregon, United States of America; 4 Charles H. Lundquist College of Business, University of Oregon, Eugene, Oregon, United States of America; 5 School of Journalism and Communication, University of Oregon, Eugene, Oregon, United States of America; 6 Department of Landscape Architecture, University of Oregon, Eugene, Oregon, United States of America; 7 Counselling Psychology and Human Services, College of Education, University of Oregon, Eugene, Oregon, United States of America; 8 Institute of Ecology and Evolution, University of Oregon, Eugene, Eugene, Oregon, United States of America

## Abstract

What do “microbes” have to do with social equity? These microorganisms are integral to our health, that of our natural environment, and even the “health” of the environments we build. The loss, gain, and retention of microorganisms—their flow between humans and the environment—can greatly impact our health. It is well-known that inequalities in access to perinatal care, healthy foods, quality housing, and the natural environment can create and arise from social inequality. Here, we focus on the argument that access to beneficial microorganisms is a facet of public health, and health inequality may be compounded by inequitable microbial exposure.

## Abstract 2

What do microbes have to do with social equity? This Essay explores the argument that access to beneficial microorganisms is a facet of public health, and that health inequality may be compounded by inequitable microbial exposure.

## What do “microbes” have to do with social equity?

Microscopic organisms—“microbes”—are integral to our health, the natural environment, and even impact the “health” of the environments we have built. Daily, we encounter millions of particles of bacteria, fungi, and viruses, as well as archaea and protozoa, and trillions more live on and in our bodies. The way that humans organize our spatial and social infrastructure affects every aspect of life, via access to perinatal care, food, buildings, the natural environment, other members of our community, water and waste management facilities, and, in all of these ways, to microorganisms. The way microorganisms and our tissues interact is determined by early life development and the maturation of the immune system, our diet and lifestyle, and the quality of our surrounding environment. Much of the health disparity in societies, which can be attributed to a lack of access stemming from social inequity, is manifested as medical conditions, which have some relation to microorganisms or lack thereof. Thus, social inequality, which impedes access to macrobiodiversity, also impedes access to microbiodiversity and the health benefits therein.

The novel concept of “microbes and social equity” is rooted in the knowledge that we rely on the microorganisms that live in or on us and our surrounding environments to provide vital ecosystem services for growth and waste recycling. The loss, gain, and retention of microorganisms can greatly impact our health and well-being. Although it has been discussed obliquely, we have yet to create a framework by which we can make better health policy or design choices using our existing knowledge of microorganisms. The ubiquity of microbes and our reliance on them extends into many aspects of social equity, such as the impact of agriculture and industry on environmental quality and conservation or privacy concerns stemming from microbial forensics, health insurance screening, or biobanking. Here, we discuss examples of microbial interactions crucial to human health and well-being that can be impeded by social policy or lack of infrastructure and how inequitable access is driving “microbial inequality.”

## Vertical transmission and the need for adequate perinatal care

Early life is a critical time period for appropriate microbial colonization as well as immune development, and it has been demonstrated in mice that there is a “priority effect” in determining long-term microbial community structure in the gut [1]. Moreover, alterations to these foundational processes have the potential to affect multiple generations [2,3]. Although evidence for direct vertical transmission of microbes in humans is mixed [4,5], there is evidence that the prenatal environment can alter fetal microbiome composition indirectly. For example, stress in early pregnancy can alter both maternal and offspring immune function and results in an altered bacterial community and metabolic profile [5,6]. Further, the stress-related changes in the maternal gut or vaginal microbiota have the potential to impact the infant gut microbiota via exposure during birth [6].

Vaginal delivery (VD) is the primary means of exposing neonates to common human symbionts via vertical transmission from multiple maternal body sites [7]. These microbial exposures lead to improved immune monitoring [8] and are posited to prime infants for balanced host–microbe interactions and immune development [5]. Cesarean (surgical) delivery can be a life-saving procedure, but the number of elective and non–medically indicated instances are dramatically rising globally [9,10], which may result from and contribute to pregnancy complications. Further, cesarean delivery circumvents exposure to maternal microbiota from vaginal and intestinal locations, leaving infants susceptible to colonization by microorganisms from other sources, including skin and the surrounding environment [11–13].

Cesarean delivery alters the infant gut microbiota and their metabolic profile [8,12], although there is not a consensus on the longevity of this effect, as the impact is modulated by other early life microbial exposures [7,12,14]. However, alterations to microbial colonization may drive immune disturbances in infants born by cesarean delivery, increasing risk for autoimmune disorders or asthma [13], and in the United States, both of these covary with minoritized racial/ethnic status and socioeconomic disadvantage [15,16]. Indeed, low socioeconomic status (SES) is strongly tied to inflammation and a number of comorbidities [17]. Given the associations between low or altered microbial diversity, inflammation, and disease, it is presumed that exposure to a diverse microbiome early in life will lead to higher microbial diversity and better microbial tolerance in adulthood and that these complex communities will provide protective advantages for the host against infection [8,18], as well as reduction in inflammation [17].

The perinatal time period, because of maternal microbial transfer, is a clear early intervention target for public health and social equity [2,19,20] and societal economic outcomes [21]. Adequate perinatal education and healthcare are well demonstrated to reduce antenatal healthcare costs [22,23], improve maternal and offspring health and psychological well-being over their lifetimes [21,24], and improve breastfeeding rates [23,25]. However, women who are socioeconomically disadvantaged experience social barriers and stressors preventing access to prenatal care, adequate nutrition, or education [23,26]. This increases the risk for complications during and after birth and increases psychological stressors, which further worsen health outcomes [13].

Breast milk contains a diverse microbial community, which is associated with microbial composition of neonatal feces [27], although direct seeding of gastrointestinal (GI) mucosal surfaces has yet to be demonstrated. More conspicuously, breast milk contains promicrobial elements that associate with neonate microbiota, namely, human milk oligosaccharides (HMOs), which enrich for specialized bacteria in the fetal gut, such as *Bifidobacterium longum* [28], which are widely demonstrated to be an important taxonomic group for infant health [28,29]. The stool of breastfed infants contains more bifidobacteria and lactobacilli and fewer pathobionts relative to formula-fed infants [29], something that supplementing formula-fed infants with *B*. *longum* can only partially replicate [30]. Breastfeeding is protective against the development of allergies, asthma, and immune disorders and leads to fewer incidences of obesity, diarrhea, respiratory tract infection, and otitis media in infants [31,32]. Breastfeeding also associates with reduced abundance of bacteria with antibiotic resistance genes, and early termination of breastfeeding can stunt this protective effect [33]. Moreover, breastfeeding reduces postpartum depression in mothers, which may be mediated by gut microbiota [14,34].

Antenatal paid leave practices vary globally by time and rate of compensation [35], and differences in ability to take parental leave may be reflective of SES disparities [36,37]. A lack of antenatal leave reduces the likelihood and duration of breastfeeding [38], especially in low-SES households [39], which are less likely to initiate breastfeeding due to a lack of social support, inadequate care at the time of birth, and misconceptions about breastfeeding [40–42]. Providing access and increasing the duration of paid parental leave improves health outcomes for mothers and infants and increases the probability of breastfeeding [42,43], thus ensuring beneficial maternal microbial transfer.

## The gut microbiome and access to adequate nutrition

Variation in diet has been linked to variation in the gut microbiota of humans [44,45], with low food diversity and fiber-poor diets (e.g., the Western diet) reducing gut microbial diversity and functionality [46]. The percentage of overweight and obese individuals has skyrocketed globally since 1975 [47]. Obesity creates comorbidities, as well as financial and social burdens [48], which lead to a lifetime decrease in SES for women [49] and is compounded by lack of education or minority status [48]. Although causative factors are complex, current evidence ties low gut microbial diversity to obesity risk [50]. A low-fiber diet is associated with the proliferation of microorganisms that are extremely efficient at extracting energy from simple fats and sugars, leaving the microbiome maladapted to metabolizing complex nutrients found in whole foods [51,52]. Moreover, experimental work supports the idea that much of our nutritional acquisition is microbially driven: germ-free mice given a fecal microbial transplant from conventional mice dramatically increased in adiposity without a significant increase in food consumption or reduction in energy expenditure [50].

Diminished gut microbial diversity is also associated with several psychiatric disorders, notably, anxiety, depression, and schizophrenia [53–55]. Neurotransmitters (neural signaling molecules) affect brain activity, learning capacity, alertness, and mood. They can be produced from dietary proteins, with a nutritious diet increasing production, but are also produced by gut microorganisms [56–58]. Bacterial dysbiosis affects the production of serotonin and gamma-aminobutyric acid (GABA) in the gut, neurotransmitters critical for regulating mental activity [53]. Germ-free mice produce fewer neurotransmitters and their precursors and exhibit psychological and cognitive changes [57,58]. Mice who received fecal transplants from human patients with schizophrenia exhibited hyperactivity, increased startle response, and depressive behavior [54].

Poor diet, especially if low in fiber (which results in low short-chain fatty acid production), may not recruit an optimum gut microbiota, and this can have a permanent impact on an individual’s neurological and mental processes [53,55]. Over one-fifth of global total healthcare burdens result from mental disorders [59], and their treatment and recovery rates are disproportionately low. Though correlations between low microbial diversity and mental illness have been observed in human populations, the directionality of this complex biological interplay is still unresolved. However, observational data and experimental manipulations in model systems (e.g., fecal-microbial transplant (FMT)s in mice) suggest that integrating dietary or lifestyle alterations designed to recruit health-associated microbes, in addition to psychiatric and psychological care, could offer additional options for mental health treatment [55]. Although pharmaceutical methods are effective and often necessary, a microbial approach may offer nutrition-based care options to those resistant to or unable to access medication or therapy [60,61].

Lower-income communities have a higher prevalence of high-fat, high-sugar, or highly processed diets, with fewer dietary options, as this food is often cheaper and more accessible [62,63]. By providing universal access to healthy foods that promote microbial diversity, diet interventions may provide an effective way to prevent the health problems associated with inadequate microbial diversity, as well as make nutritional access more equitable [20,46,52]. Importantly, eliminating food deserts is a way to improve public health by reducing the prevalence of obesity, as well as other nutrition-associated health problems [52,64,65], and may also reduce health problems associated with low microbial diversity. School lunch programs that provide food and exclude other unhealthy foods and beverages improve nutrition standards [66] and student learning [67]. More broadly, requiring grocery stores to carry fresh fruits and vegetables [68], financial incentives or assistance to small groceries in food deserts [69,70], or food assistance programs have all been shown to improve access to healthy food [65].

## Microbiology of the built environment and spatial justice

Water damage and building deterioration contribute to indoor air pollution and accrual of microorganisms, often making the space unsuitable for occupants [71], something that disproportionately affects low-income populations [72,73]. Many schools or other public infrastructure buildings contain high microbial biomass in the air and on surfaces [71,74], which can also disproportionately affect people of lower SES [75,76]. Similarly, very little infrastructure or policy considers microorganisms in prisons, as evidenced by a lack of hand-washing stations or showers, inadequate food service infrastructure, or difficulty in cleaning or quarantining areas [77]. Overcrowding overwhelms sanitation efforts, and increased proximity promotes the transmission of contagious agents, many of which are effectively endemic [77–80]. These conditions indicate either a lack of attention to the microbial health of prison facilities and their occupants or, more likely, a lack of priority on equitable care [79,81,82].

On average, 55% of the current global population resides in cities [83]. Living in an urban environment directly reduces microbial exposure [84,85]. Yet there is increasing evidence that exposure to diverse microbiota, including outdoor-sourced microorganisms from soil, water, and plants, is integral to our health [84,86]. Environmental microbial exposure promotes immune signaling and helps build adaptive immunity [84] and is associated with reduced rates of certain infectious diseases [87,88] or asthma and allergies [89,90]. Furthermore, exposure to air pollution has been directly linked to gut microbiota disorder and inflammatory bowel disease (IBD) prevalence [91].

Urban soils and waters exhibit spatial variation in their microbial communities based on green infrastructure type, soil composition, plant biodiversity, and size [92,93] and provide exposure to increased microbial diversity, with the potential to combat microbial loss from urbanization [84]. To manage urban stormwater, many areas are implementing above-ground “green” strategies, which employ plants and soils to control the speed, volume, temperature, and quality of drainage. With vegetation, soils, and sporadic standing water, this green infrastructure functions as small-scale parks and provides habitat for complex microbial communities [94]. The distribution of these amenities themselves has implications for equity (i.e., spatial justice), because such facilities often accompany redevelopment projects or new development rather than older neighborhoods.

Zoning partitions land by use and intends to foster public health by physical separation of residence space from industry and pollution [95], yet inequitable zoning creates neighborhoods with unequal exposure to environmental risks or benefits and can lead to large-scale public health disparities [96–99]. Studies suggest that pollution-heavy industry is intentionally placed in disadvantaged neighborhoods [97,98]. Zoning and policy could be used to aid in the equitable distribution of resources [98]: supporting urban farms and local farmers’ markets, improving clean water and waste management facilities, reducing exposure to industrial pollution, applying conditional-use permits to require stores to offer healthy food items, or distributing greenspace and environmental microorganisms equitably.

## Do we have a right to microbes?

The importance of microorganisms to biological life is evident; their presence provides the foundation for our own cellular complexity and the very environment on which we depend [100,101]. The question of whether we own our microbiota and whether we have the right to microbiota is central to the argument of microbiota as a means of social equity because of their vital role in our health and development. Ownership of biological tissue is a legal “grey area” [102], but the sale of bodily fluids or byproducts, including microorganisms, is generally legal [103]. We cannot say we own our microbiota in the way that we have an innate right to own our biological tissues [102]; microorganisms are too intransigent for that. If we do not own them, per se, then perhaps we have a right to access and use microorganisms, much in the way that we have a right to access natural environments and the publicly shared environmental resources we require to live [104].

The advent of microbially based therapeutics (i.e., probiotics) has opened the door to commercial early adopters peddling presumptive “healthy microbes” [105]. It has also added a new component to “biobanking”—the practice of archiving biological material—and the question of “who owns your poop” has been discussed [103,106] in this new age of fecal-prospecting for medical therapeutics. Much of this discussion regards privacy protection, as even fecal samples carry human cells tagged with our genetic information. However, it brings up yet another question regarding access. If we consider microorganisms to be “collectively owned resources,” do we not collectively have the right to benefit from microorganisms and the metabolites they produce?

Access is the basis for creating and resolving social equity—access to healthcare, healthy foods, a suitable environment, and now, those microorganisms that are demonstrated to be altered by the lifestyle differences inherent to social inequity and lack of access to a variety of resources. If governments have a legal obligation to provide access to a healthy natural environment, and if microbial communities are integral to maintaining public health, it follows that there is likewise a legal obligation to provide policy and infrastructure to enable equitable access to microorganisms. The health, social, and financial benefits of supplying social welfare programs that provide healthcare, food, and shelter—and, in particular, those that benefit people who are marginalized and lacking in resources—are well demonstrated [22,107,108]. Even without an understanding of the effect of microorganisms on our lives, it is recognized that individual health and well-being is a common good. As our knowledge of the integral role that microorganisms play in our lives grows, we come to understand that social and political barriers to the resources required to maintain our microbiome also become an issue of social equity.

## Abbreviations

FMT: fecal-microbial transplant
GABA: gamma-aminobutyric acid
GI: gastrointestinal
HMO: human milk oligosaccharide
IBD: inflammatory bowel disease
SES: socioeconomic status
VD: vaginal delivery
